# Reversing Preeclampsia Pathology: AXL Inhibition Restores Mitochondrial Function and ECM Balance

**DOI:** 10.3390/cells14161229

**Published:** 2025-08-08

**Authors:** Archarlie Chou, Benjamin Davidson, Paul R. Reynolds, Brett E. Pickett, Juan A. Arroyo

**Affiliations:** 1 Department of Microbiology and Molecular Biology, Brigham Young University, Provo, UT 84602, USA; 2 Department of Cell Biology and Physiology, Brigham Young University, Provo, UT 84602, USA

**Keywords:** preeclampsia, Gas6, AXL inhibition, RNA seq, placenta

## Abstract

Preeclampsia (PE) is a leading cause of maternal and fetal morbidity that affects 2–8% of pregnancies worldwide, driven by placental dysfunction and systemic inflammation. Growth arrest-specific protein 6 (Gas6) and its receptor AXL play pivotal roles in PE pathogenesis, promoting trophoblast impairment and vascular dysregulation. This study investigated the transcriptomic reversal effects of AXL Receptor Tyrosine Kinase (AXL) inhibition in a Gas6-induced rat model of PE using RNA sequencing (RNA-seq). Pregnant rats were administered Gas6 to induce PE-like symptoms such as hypertension and proteinuria; a subset also received the AXL inhibitor R428. RNA-seq of placental tissues revealed 2331 differentially expressed genes (DEGs) in Gas6-AXLi versus Gas6 (1277 upregulated, 1054 downregulated). Protein–protein interaction networks and Gene Ontology enrichment highlighted upregulated mitochondrial functions, including electron transport chain components (e.g., NDUFC2, COX5A), suggesting enhanced energy metabolism. In the secondary analysis that compared Gas6 to Control, Gas6-upregulated extracellular matrix proteins (e.g., COL4A1, LAMC1) linked to fibrosis were reversed by AXL inhibition, indicating ameliorated placental remodeling. AXL inhibition activated compensatory pathways beyond Gas6 blockade, unveiling novel mechanisms for PE resolution. These findings position AXL inhibitors as promising therapeutics, offering insights into mitochondrial and fibrotic targets to mitigate this enigmatic disorder.

## 1. Introduction

Preeclampsia (PE) remains an ongoing challenge in maternal–fetal medicine as one of the leading causes of maternal and neonatal morbidity and mortality globally. Defined by new-onset hypertension after 20 weeks of gestation, this disease is often accompanied by proteinuria or other signs of organ dysfunction. PE affects approximately 2–8% of pregnancies and is implicated in over 50,000 maternal deaths annually [1,2]. Beyond its immediate threats, such as preterm delivery, fetal growth restriction, and maternal organ failure, PE casts a long shadow that predisposes both mother and child to future health risks, including cardiovascular disease, hypertension, and metabolic disorders [3].

Central to the pathogenesis of PE is placental dysfunction, which disrupts the delicate maternal–fetal interface and triggers a cascade of systemic maternal responses, including endothelial dysfunction, chronic inflammation, and oxidative stress [4]. The placenta orchestrates fetal nourishment and maternal adaptation through intricate cellular processes, particularly via trophoblast cells responsible for spiral artery remodeling and placental invasion [5]. In PE, impaired trophoblast function leads to inadequate placental perfusion, hypoxia, and the release of pro-inflammatory and anti-angiogenic factors into the maternal circulation [6]. 

Beyond its role in placental biology, Gas6 is implicated in various human pathologies. It functions as a ligand for the TAM family of receptor tyrosine kinases (Tyro3, AXL, MerTK) and is known to regulate inflammation, coagulation, immune homeostasis, and tissue remodeling. In cancer, overactivation of the Gas6/AXL pathway promotes tumor cell survival, migration, epithelial–mesenchymal transition (EMT), and resistance to therapy. Additionally, Gas6/AXL signaling contributes to fibrotic diseases (e.g., pulmonary and renal fibrosis), autoimmune conditions such as systemic lupus erythematosus, and thrombotic disorders due to its role in platelet activation and vascular inflammation. These diverse roles highlight Gas6 as a critical modulator of pathophysiological processes in both immune and vascular systems.

Prior transcriptomic and proteomic studies have reported the presence of disruptions in both the electron transport chain (ETC) and excessive oxidative stress during disease [7,8,9]. Briefly, NADH and FADH_2_ are generated during the Krebs’s cycle, contributing to maintaining a proton gradient in complex I of the ETC. Dysfunction in NADH dehydrogenase (a member of Complex I) can cause electron leakage in the inner mitochondrial membrane space, where electrons escape and react prematurely with molecular oxygen, forming superoxide, further damaging the cells. Prior studies have also reported that Complex IV and Complex I are downregulated in PE [10].

Besides damaging the ETC, another common sign associated with PE is the thickening of the placental decidua layer [11]. The human placenta secretes collagen types I, III, IV, V, and VI [12,13]. However, excessive collagen deposition in human PE increases tissue rigidity, impairing trophoblast invasion [14,15].

Given these placental structure and function abnormalities, researchers have turned to cancer biology for clues. The Gas6/AXL axis has gained attention for its roles in invasion. The growth arrest-specific 6 (Gas6) protein is a receptor tyrosine kinase ligand dependent on vitamin K to activate AXL. This binding mechanism regulates diverse cellular functions, including cell survival, migration, and immune modulation [16]. Gas6/AXL signaling is critical for trophoblast differentiation and vascular remodeling in the placenta. Still, its dysregulation in PE has been linked to impaired trophoblast invasion, heightened oxidative stress, and excessive inflammation [17]. Elevated levels of Gas6 and soluble AXL in maternal plasma have been observed in women with severe PE, which correlate with markers of endothelial dysfunction and systemic inflammation [17,18,19,20]. These findings position Gas6/AXL signaling as a pivotal axis in PE pathophysiology, with potential to be both a biomarker and therapeutic target.

This study employed bulk RNA-sequencing to interrogate the transcriptomic landscape of Gas6/AXL-induced PE in a rat model. By integrating controlled Gas6 administration with AXL inhibition and high-throughput RNA sequencing, we aimed to delineate the gene expression profiles and biological processes modulated by Gas6/AXL signaling, and to validate their relevance to human PE.

## 2. Materials and Methods

### 2.1. Animals and Tissue Collection

This study was approved by the Brigham Young University Institutional Animal Care and Use Committee (IACUC; Approval number PRE21-0012). Holtzman Sprague Dawley (HSD) rats (30; ~400 g) were subjected to experimental protocols approved by the IACUC at Brigham Young University [17]. Timed pregnancies were established by overnight mating, with gestational day 0.5 confirmed via sperm-positive vaginal smears. On gestational day 18.5 (dGA), necropsy was performed. Placental samples were snap-frozen in liquid nitrogen and stored at −80 °C for subsequent RNA analysis.

### 2.2. Animal Treatment Protocols

**Figure 1:**
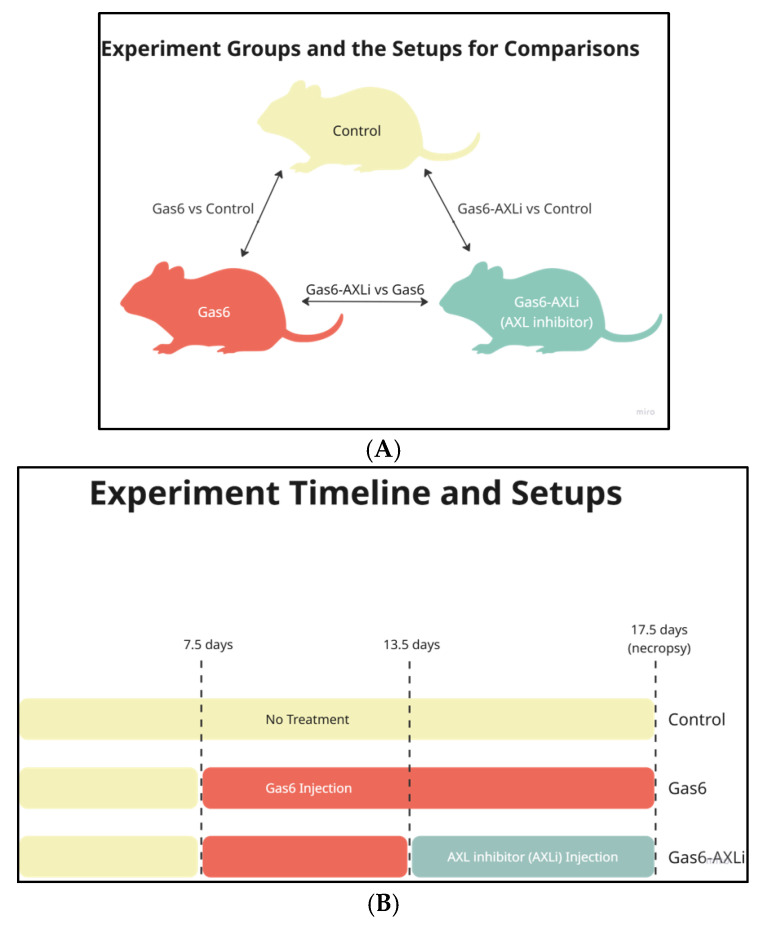
Experimental Design and Treatment Timeline for Mouse Comparisons: (**A**) Illustration of mouse treatments and the comparison groups; (**B**) Timeline diagram of the experiment comparing treatment (Gas6, Gas6-AXLi) and control groups.

To induce a PE-like phenotype, pregnant rats (n = 10) received daily intraperitoneal (i.p.) injections of Gas6 (4 µg/kg, R&D Systems) from dGA 7.5 to 17.5. A control group (n = 10) received saline injections and was pair-fed to match the rats treated with Gas6 intake. A third group (n = 10) received the same Gas6 treatment in combination with the AXL inhibitor R428 (75 mg/kg/day, APExBIO) from dGA 13.5 to 17.5, a window corresponding to mid-gestation when PE features typically emerge (Figure 1A,B). These groups were designated Control, Gas6, and Gas6-AXLi, respectively (Figure 1A). Blood pressure was measured daily using the CODA tail-cuff system, a validated non-invasive quantitative technique for conscious rodents. Proteinuria was assessed at necropsy using a dipstick method, with levels of +3 (300 mg/dL) or +4 (≥2000 mg/dL) considered indicative of PE.

### 2.3. RNA Isolation and Extraction

Total RNA was extracted from placental tissue using the Direct-zol RNA MiniPrep Plus kit and TriReagent (Zymo Research, Irvine, CA, USA), including on-column DNase I digestion. RNA integrity was preserved through standardized ethanol precipitation, column purification, and elution in RNase-free water. Polyadenylated mRNA was enriched using magnetic oligo (dT) beads, followed by cDNA synthesis, end repair, A-tailing, Illumina adapter ligation, size selection, PCR amplification, and purification. Libraries were sequenced as 150-bp paired-end reads on an Illumina NovaSeq 6000 instrument Illumina, San Diego, CA, USA as previously described [21,22].

### 2.4. RNA Sequencing Data Analysis

Preprocessing of the bulk RNA-sequencing reads was performed using the RASflow pipeline [23]. Briefly, this workflow carries out the following steps: quality control with FastQC (https://www.bioinformatics.babraham.ac.uk/projects/fastqc/, accessed on 3 July 2025), read trimming with TrimGalore (https://www.bioinformatics.babraham.ac.uk/projects/trim_galore/, accessed on 3 July 2025), read mapping and quantification with Salmon [24], mapping of transcripts to genes with tximport, and differential expression analysis with edgeR with negative binomial statistics (v4.6.2) [25] for the two conditions: (1) Gas6-AXLi vs. Gas6 and (2) Gas6-AXLi vs. Control. The list of statistically significantly differentially expressed genes (DEGs) was produced using an FDR-adjusted *p*-value < 0.05 as the threshold. The DEGs were mapped from *Rattus norvegicus* Ensembl IDs (RGSC3.4; rn4 release 52) to their corresponding human Ensembl IDs using Biomart.

A protein interaction network with all the DEGs (FDR < 0.05) was constructed using human interaction data from the STRING database [26] (edge confidence score ≥ 900); (9606.protein.actions.v10.5) to improve downstream analysis and interpretation for the large number of DEGs. Only the following STRING action modes were included in this analysis to improve accuracy and interpretation: (1) Activation, (2) Inhibition, (3) Reaction, and (4) Binding. The network graph was then computationally subdivided into 21 smaller subgraphs based on topology using the function ‘greedy_modularity_communities’ with all default parameters from the NetworkX Python library (https://www.osti.gov/biblio/960616-V3.5, accessed on 3 July 2025) The function utilized Clauset–Newman–Moore greedy modularity maximization [27], a method developed to find community partitions in interaction networks with the largest modularity.

These subgraphs facilitated the following tasks: (1) reducing the size of the complete network, and (2) facilitating more precise Gene Ontology (GO) enrichment analysis with hypergeometric statistics [28] for each subgraph. Each subgraph was then annotated with GO terms, and an overrepresentation enrichment analysis of these terms was performed to identify those that met the threshold for statistical significance (FDR-adjusted q-value < 0.0001). This approach enabled a more holistic interpretation of the underlying known function(s) for the interactions in each subgraph. After calculating the significant GO terms for each subgraph, a heatmap was generated to display the log2 fold change values of the involved DEGs for each term and their corresponding expression across the other comparisons. This enabled improved interpretation of the regulation of a biological process based on its gene expression.

In the secondary analysis of Gas6 vs. Control, complementary GO enrichment strategies were implemented due to the lower number of DEGs, which rendered sub-graphing unnecessary. The DEGs (FDR < 0.05) were first divided into upregulated DEGs and downregulated DEGs. Statistical enrichments were then calculated for each of the three branches of the Gene Ontology: biological process (BP), cellular component (CC), and molecular function (MF) enrichment (q-value < 0.001) using the overrepresentation method on both the upregulated and downregulated DEGs.

### 2.5. Statistical Analysis

Differences in proteinuria and blood pressure were assessed for statistical significance via an unpaired Student’s *t*-test, with a threshold of *p* < 0.05. For the RNA-sequencing dataset, differential gene expression was evaluated using a negative binomial distribution model in the edgeR package [19], followed by false discovery rate (FDR) adjustment to account for multiple comparisons (FDR < 0.05). Gene Ontology (GO) enrichment analysis was implemented with hypergeometric tests. The Clauset–Newman–Moore greedy modularity maximization algorithm was used to produce the subgraphs.

## 3. Results

### 3.1. Gas6 Preeclampsia and AXL Inhibition

**Figure 2:**
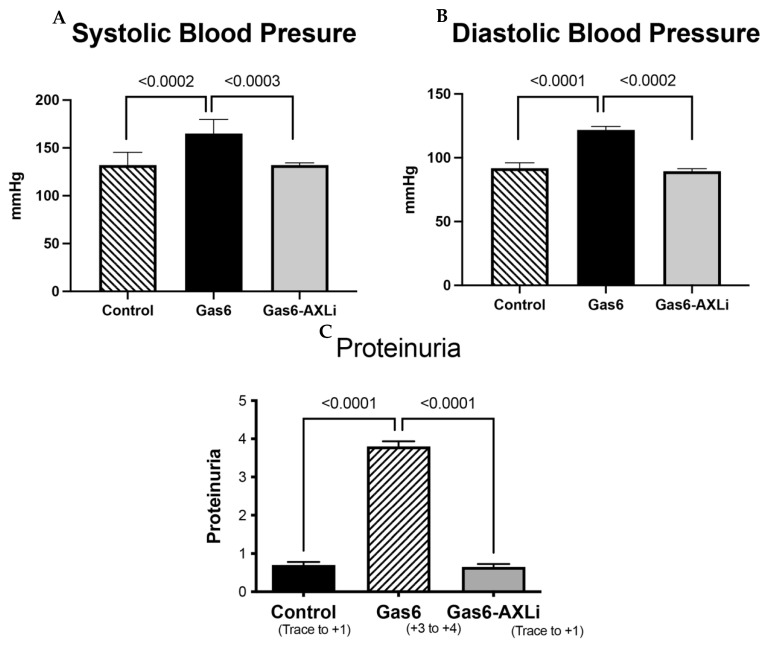
Blood Pressure and Proteinuria in Control and Treated Animals. A significant increase in both systolic and diastolic pressure was induced by Gas6 treatment but significantly decreased when AXL was inhibited (**A**,**B**). Gas6-induced proteinuria was reduced with AXL inhibition (**C**).

We first confirmed the presence of various well-established signs and symptoms of PE, including high blood pressure and proteinuria, in the animal models. We observed that Gas6 treatment significantly increased systolic and diastolic blood pressure in the rats, as measured at necropsy (Figure 2C). This increase was reduced when AXL was inhibited by R428 (Figure 2A,B). Similarly, we found that proteinuria was markedly increased (+3 to +4) in Gas6-treated rats compared to the healthy control group and reduced to basal levels when pregnant animals were treated with Gas6 and the AXL inhibitor (Figure 2C).

### 3.2. Differentially Expressed Genes (DEGs) Analysis

**Figure 3:**
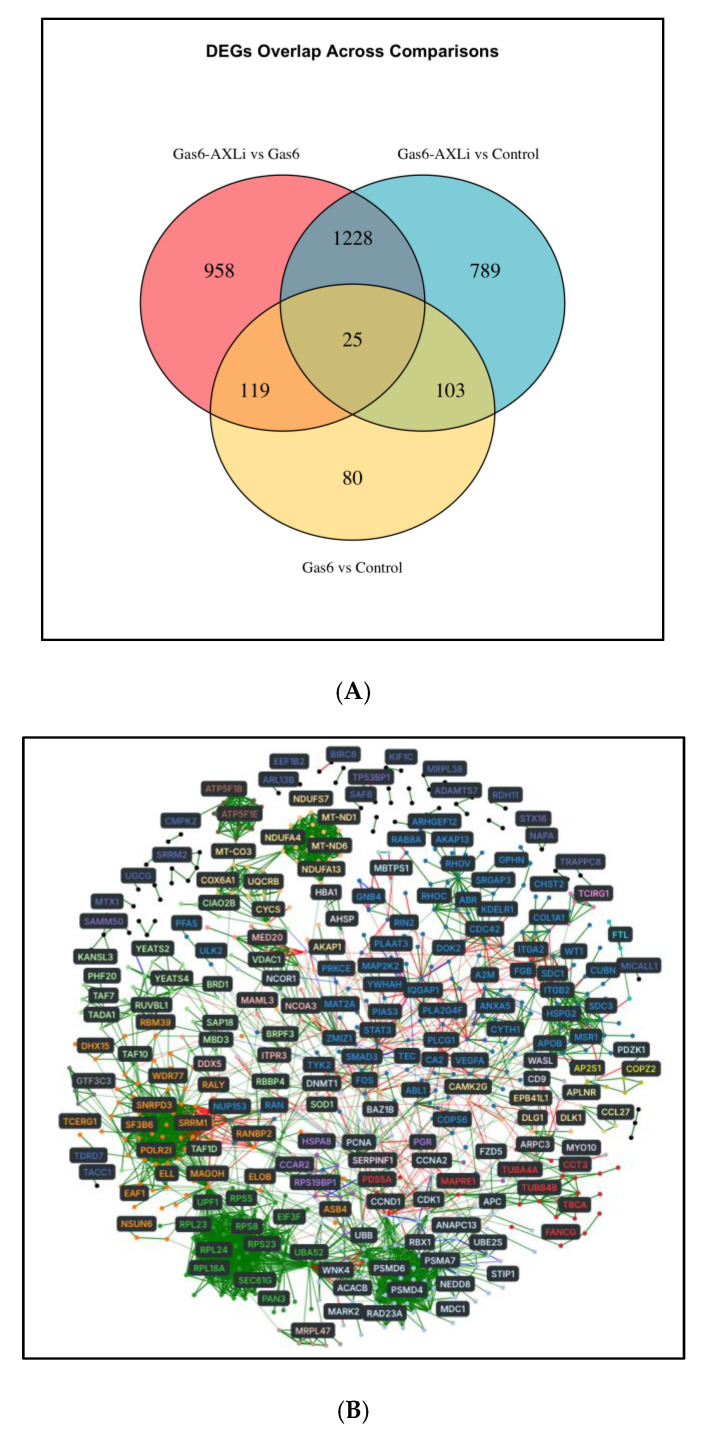
Overlapping DEGs and Topological Clustering of Protein Interactions Across All Comparisons. (**A**) Overlapping DEGs across the three distinct comparisons (186 genes total) are represented in a Venn diagram (FDR < 0.05, |logFC| > 1.5). (**B**) The PPI network was constructed using public STRING protein–protein interactions. Nodes (proteins) in each network are colored to represent the 21 unique subgraphs or communities to which they belong. Color of the edges—Red: activation, Blue: inhibition, Green: interaction or binding.

To identify transcriptomic differences across conditions, we compared DEGs between groups and summarized the overlapping genes using a Venn diagram (FDR < 0.05, |log_2_FC| ≥ 1.5) (Figure 3A). The Gas6 vs. Gas6-AXLi comparison had the most DEGs (n = 2331), while the Gas6-AXLi vs. Control comparison had a moderate number (n = 2145), and the Gas6 vs. Control comparison yielded the fewest DEGs (n = 327).

### 3.3. Network and Gene Ontology (GO) Analysis of Gas6-AXLi vs. Gas6

We next wanted to understand better the functional roles of significant DEGs (FDR < 0.05) within the PPI network. For the Gas6-AXLi vs. Gas6 comparison, we first mapped all rat DEGs to their corresponding human orthologs and used the STRING database to construct a PPI network based on known interactions. We deconstructed this PPI network, using the NetworkX algorithm, and observed 21 subgraphs (communities) (Figure 3B). We then performed GO enrichment analysis on each community to identify its enriched annotated biological functions. This approach enabled the grouping of interacting genes into functionally coherent modules, allowing for a more structured and less biased interpretation of the network with a finer level of granularity.

### 3.4. Mitochondrial Functions (Gas6-AXLi vs. Gas6)

**Figure 4:**
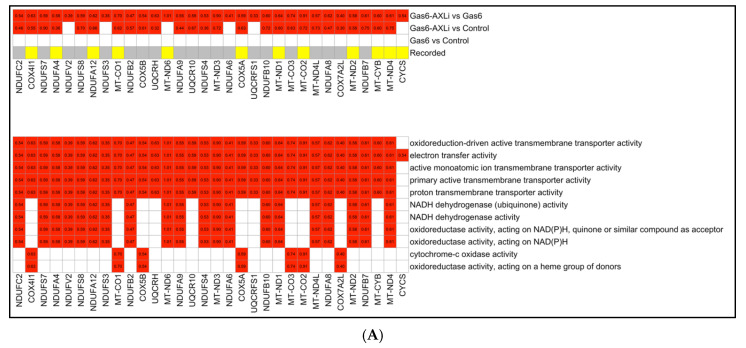

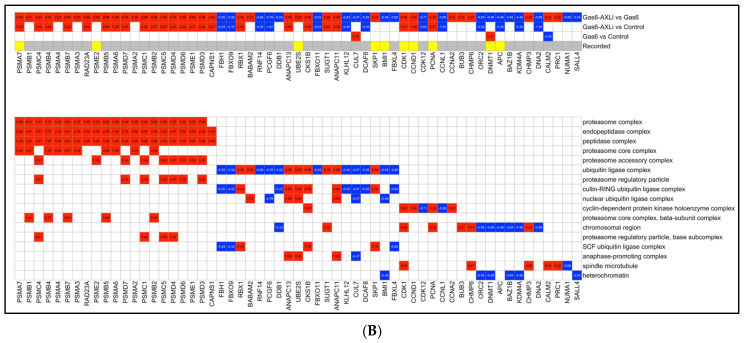
Detailed GO Terms Annotated with Gene Expression from All Groups. (**A**) Top heatmap showing the gene expression across three different comparison groups. Bottom heatmap showing gene expression across three different comparisons. In the fourth row (recorded), colors indicate whether known gene-disease associations exist in the literature (yellow) or not (gray). (**B**) Top heatmap showing the gene expression across three different comparison groups. Bottom heatmap displaying the individual upregulated (red) or downregulated (blue) genes annotated with each enriched GO term. The log_2_FC values are displayed inside each square.

Following AXLi treatment, we found numerous significantly upregulated genes associated with mitochondrial function, potentially indicating either a restoration of mitochondrial metabolism or a redundant mechanism that results in the same outcome (Figure 4A). We also observed that key components of the ETC exhibited consistent transcriptional activation in the Gas6-AXLi vs. Gas6 comparison.

We found that various Complex I ETC gene products were upregulated, including NDUFC2 (log_2_ fold changes = 0.54, *p* = 0.0002), NDUFS7 (log_2_FC = 0.59, *p* = 0.005), NDUFA4 (log_2_FC = 0.58, *p* = 1.00 × 10^−6^), NDUFV2 (0.39, *p* = 0.014), and NDUFS8 (0.59, *p* = 0.0002). These components support ATP production and reduce ROS production. Similarly, we observed that genes encoding Complex III subunits were also upregulated, including UQCRH (0.83, *p* = 2.64 × 10^−9^), UQCR10 (0.59, *p* = 3.58 × 10^−7^), and UQCRFS1 (0.33, *p* = 0.003), as well as Complex IV genes, COX4I1 (0.63, *p* = 1.55 × 10^−9^), COX5A (0.59, *p* = 0.00019), COX5B (0.54, *p* = 1.52 × 10^−6^), COX7A2L (0.40, *p* = 1.9 × 10^−4^), MT-CO1 (0.70, *p* = 2.46 × 10^−14^), MT-CO2 (0.91, *p* = 4.69 × 10^−19^), and MT-CO3 (0.74, *p* = 4.69 × 10^−19^). This observation was interesting since these gene products facilitate electron transfer, increase oxygen consumption, and enhance mitochondrial energy output. Upregulation of mitochondrial DNA-encoded genes, such as MT-ND1 (0.64, *p* = 8.54 × 10^−9^) and MT-CO1 (0.70, *p* = 2.46 × 10^−14^), was observed. Additionally, elevated expression of CYCS (0.54, *p* = 2.75 × 10^−9^), which encodes cytochrome C, suggests enhanced ETC activity.

### 3.5. Upregulation of Proteasome and Ubiquitin Ligase Complex (Gas6-AXLi vs. Gas6)

Our analysis also identified 21 proteasome subunit genes that were uniformly upregulated, indicating enhanced regulation of proteolysis (Figure 4B). Among the upregulated 20S core alpha subunits, PSMA7 (0.36, *p* = 0.004), PSMA4 (0.34, *p* = 0.005), PSMA3 (0.35, *p* = 0.003), PSMA6 (0.45, *p* = 5.74 × 10^−6^), and PSMA2 (0.36, *p* = 0.001) are essential for substrate recognition and binding. The 20S core beta subunits PSMB1 (0.41, *p* = 0.005), PSMB4, PSMB7 (0.61, *p* = 2.67 × 10^−8^), and PSMB2 contain the catalytic sites responsible for protein degradation. PSMA subunits also showed higher protein expression compared to the control group at the endpoint. PSME1, a component of the 11S regulatory complex (PA28), was upregulated. RAD23A (0.48, *p* = 0.001), a ubiquitin-binding shuttle protein, was also upregulated and likely facilitates the delivery of ubiquitinated proteins to the proteasome. Genes associated with the ubiquitin ligase complex were also enriched, although their expression patterns were less consistent.

### 3.6. Gas6 vs. Control Secondary Analysis: Reversal of Collagen Expression

To comprehensively assess the effects of potential reversal of AXL signaling via AXLi treatment, we conducted a secondary analysis on the Gas6 vs. Control comparison. The focus of this analysis was to identify expression patterns in this comparison against those observed in the Gas6-AXLi vs. Gas6 and Gas6-AXLi vs. Control comparisons.

**Figure 5:**
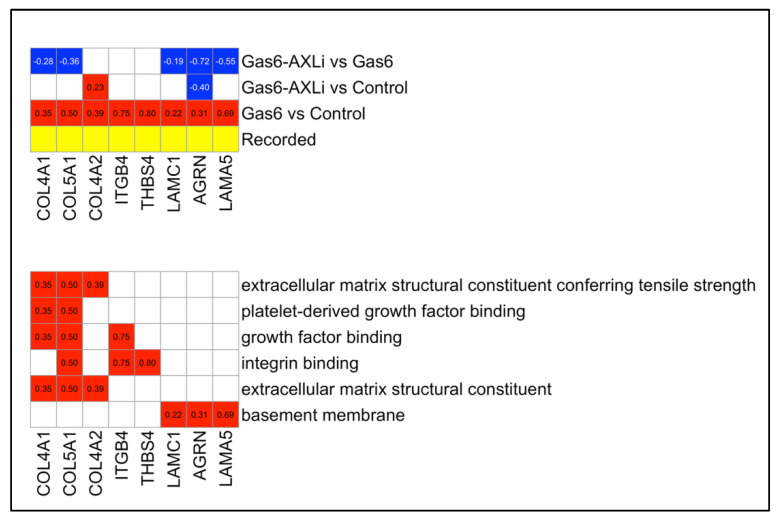
Expression Across All Comparisons. Top heatmap showing the gene expression across three different comparison groups, with detailed ECM-associated GO terms annotated with eight differentially expressed genes. Bottom heatmap displaying the same eight ECM-related differentially expressed genes that were significantly enriched for six GO terms in this community. All eight genes were upregulated with Gas6 treatment (Gas6 vs. Control), while a subset was downregulated following AXLi treatment. Color scheme is identical to Figure 4.

Several ECM-related genes were significantly upregulated following Gas6 treatment (Gas6 vs. Control) and subsequently downregulated with AXLi treatment (Gas6-AXLi vs. Gas6), suggesting a reversal of Gas6-induced transcriptional activation (Figure 5). These include LAMC1 (Gas6: 0.22, *p* = 0.032; AXLi: −0.19, *p* = 0.025), LAMA5 (0.69, *p* = 5.68 × 10^−13^; AXLi: −0.55, *p* = 1.36 × 10^−7^), AGRN (0.31, *p* = 0.01; AXLi: −0.72, *p* = 8.33 × 10^−14^), COL4A1 (0.35, *p* = 0.012; AXLi: −0.28, *p* = 0.01), and COL5A1 (0.50, *p* = 0.024; AXLi: −0.36, *p* = 0.025).

In contrast, a subset of ECM genes remained elevated despite AXLi intervention, indicating persistent upregulation. These include COL4A2 (Gas6: 0.39, *p* = 0.015; AXLi: −0.16, *p* = 0.27), ITGB4 (0.75, *p* = 3 × 10^−4^; AXLi: −0.39, *p* = 0.12), and THBS4 (0.80, *p* = 0.024; AXLi: 0.07, *p* = 0.93) (Figure 5). These genes are involved in ECM organization and vascular remodeling, pathways that are relevant to the endothelial dysfunction observed in PE.

### 3.7. Gas6 vs. Control Secondary Analysis: Lipid Metabolism and Ubiquitin-Associated Functions

Our observed upregulation of mitochondrial genes with AXLi treatment prompted us to investigate energy-related processes in the context of Gas6-induced PE. Our previous research indicated impaired mitochondrial function following Gas6 treatment, motivating a more in-depth analysis. To do this, we divided the DEGs from the Gas6 vs. Control comparison into upregulated and downregulated groups and performed GO enrichment analysis separately on each group.

**Figure 6:**
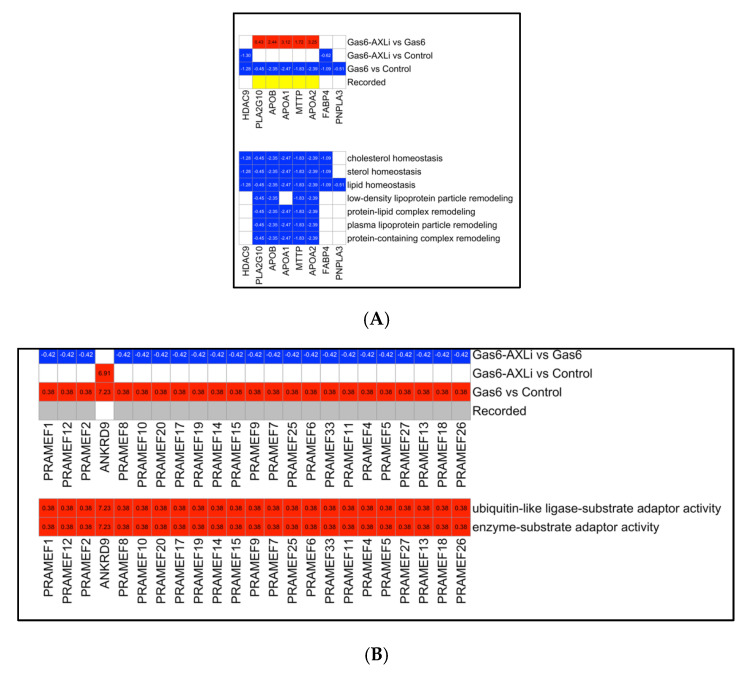
GO Term Enrichment of Differentially Expressed Genes with PE Association Annotations. (**A**) Top heatmap showing the gene expression across three different comparison groups. Bottom heatmap showing downregulated genes enriched in Gas6 vs. Control, identifying eight genes that were significantly enriched with seven GO terms. (**B**) Top heatmap showing the gene expression across three different comparison groups. Bottom heatmap showing 23 preferentially expressed antigen of melanoma (PRAME) genes that were significantly enriched with two GO terms. Color scheme is identical to Figure 4.

The GO analysis of downregulated genes revealed multiple lipid-associated biological processes, including lipid homeostasis, low-density lipoprotein particle remodeling, and protein–lipid complex remodeling (Figure 6A). Key lipid metabolism genes such as HDAC9 (−1.28, *p* = 0.024), PLA2G10 (−0.45, *p* = 0.033), APOB (−2.35, *p* = 0.038), APOA1 (−2.47, *p* = 7.53 × 10^−4^), MTTP (−2.47, *p* = 0.001), APOA2 (−2.39, *p* = 0.049), FABP4 (−0.01, *p* = 1 × 10^−4^), and PNPLA3 (−0.51, *p* = 0.026) were downregulated following Gas6 treatment when compared to the mock-treated control. After AXLi treatment, expression of most of these genes returned to control levels, except for HDAC9 (−1.28, *p* = 0.024), FABP4, and PNPLA3, which remained suppressed.

Conversely, GO analysis of the upregulated genes showed strong enrichment for ubiquitin-associated functions (Figure 6B). Notably, all 23 PRAME family genes were upregulated in response to Gas6 treatment but returned to control expression levels after AXLi treatment. The only exception was ANKRD9 (7.23, *p* = 4.37 × 10^−8^), which remained consistently upregulated across all conditions.

### 3.8. Reversal of Gas6-Induced Upregulation

**Figure 7:**
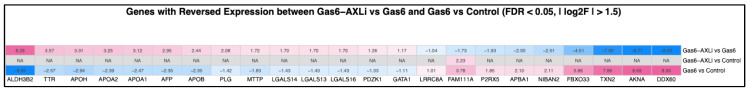
Genes with Reversed Expression between Gas6-AXLi vs. Gas6 vs. Control. The color gradient depicts the up (red) or down (blue) regulation of the genes. Only genes with direct reversal in regulation are included. (FDR < 0.05, |log2F| > 1.5).

A subset of genes upregulated in Gas6-treated rats returned to control levels or were downregulated following AXLi intervention (Figure 7, Table 1). These included ALDH3B2, TTR, APOA1, APOH, APOA2, APOB, AFP, PLG, MTTP, LGALS14, LGALS13, LGALS16, PDZK1, and GATA1. This group is primarily involved in regulating oxidative stress, lipid transport, coagulation, immune modulation, and trophoblast function.

### 3.9. Reversal of Gas6-Induced Downregulation

Conversely, several genes that Gas6 downregulated became upregulated or returned to baseline following AXLi treatment. These include LRRC8A, FAM111A, P2RX5, APBA1, NIBAN2, FBXO33, TXN2, AKNA, and DDX60. These genes are associated with cellular homeostasis, mitochondrial activity, transcriptional control, and innate immune defense.

LRRC8A, a volume-regulated anion channel, exemplified this pattern of reversal. FAM111A, which encodes a DNA replication-associated protease involved in cellular stress responses, also showed increased expression after AXLi. Although levels declined relative to Gas6, they remained elevated compared to the control group. TXN2, a mitochondrial thioredoxin essential for the detoxification of reactive oxygen species, was similarly induced. Additionally, AKNA, a transcription factor critical for fertility and immune regulation, and DDX60, an interferon-inducible RNA helicase implicated in placental innate immunity, were upregulated only after AXLi treatment. These expression patterns support a possible role for these genes in post-reversal compensation and restoration of placental function.

## 4. Discussion

The current study elucidates the transcriptomic effects of AXL inhibition in a Gas6-induced rat model of PE, revealing mechanisms by which pharmacological blockade of the Gas6/AXL pathway ameliorates PE-like symptoms. We observed that administering Gas6 to pregnant rats induced hallmark features of PE, including elevated systolic and diastolic blood pressure and proteinuria, consistent with prior evidence that Gas6/AXL signaling drives placental dysfunction and systemic vascular changes [17]. Notably, co-treatment with the AXL inhibitor R428 appeared to reverse these clinical manifestations, aligning with its ability to mitigate platelet aggregation and endothelial dysfunction in this pathway. RNA-seq analysis identified 2331 DEGs in the Gas6-AXLi versus Gas6 comparison, with 1277 upregulated and 1054 downregulated. PPI network clustering and GO enrichment show enhanced mitochondrial activity, reduced ECM deposition, and modulated proteasome–ubiquitin pathways.

### 4.1. Restoration of Mitochondrial Function by AXL Inhibition

A prominent finding was the uniform upregulation of genes associated with mitochondrial components. Mitochondrial dysfunction in PE is well-documented, often manifesting as impaired ETC activity, reduced ATP production, and elevated ROS, exacerbating trophoblast apoptosis and systemic inflammation [29]. Over the past three decades, substantial evidence links mitochondrial abnormalities to placental dysfunction in PE, with differential involvement in early- and late-onset forms [10,30,31]. In our model, Gas6 treatment likely mimics this dysfunction, as secondary analysis revealed downregulation of lipid metabolism genes affected by AXL inhibition. This reversal aligns with evidence that dyslipidemia in PE impairs mitochondrial lipid handling and contributes to placental hypoxia [30,32,33,34]. Thus, AXL inhibition may enhance mitochondrial resilience, reduce ROS, and support trophoblast survival.

### 4.2. Reduction in ECM Deposition and Fibrosis Reversal

Gas6 treatment upregulated ECM and basement membrane proteins indicative of fibrosis and impaired vascular remodeling in PE placentas. AXL inhibition appeared to reverse this pattern for most genes, suggesting amelioration of decidual thickening and placental stiffness. Fibrotic changes in PE disrupt trophoblast invasion and spiral artery remodeling, contributing to hypoxia [4]. Type I collagen replacement of Type IV collagen significantly affects trophoblast fusion efficiency and placental function [35]. Our findings imply that Gas6/AXL at least partially drives fibrotic gene expression, and that inhibition appears to restore ECM balance by potentially improving placental perfusion.

### 4.3. Modulation of Proteasome-Ubiquitin Pathways

Upregulation of 21 proteasome subunits and ubiquitin-related genes following AXL inhibition suggests enhanced protein degradation, potentially clearing misfolded proteins accumulated in PE. Downregulation of PSME3, as reported in severe PE, was observed, while upregulation of PSME1 may enhance peptide hydrolysis [36]. Ubiquitin-proteasome dysfunction in PE leads to aggregate accumulation and impaired autophagy (aggrephagy), contributing to poor placentation [37].

### 4.4. Gene Expression Patterns and Reversal Categories

The genes reversed by AXLi treatment suggest several candidate mechanisms may be involved in the development and resolution of PE. For instance, ALDH3B2, known to detoxify reactive aldehydes, was previously found to be downregulated in PE-associated mesenchymal stem cells, implicating disrupted oxidative stress handling in placental dysfunction [38]. Similarly, reduced TTR (transthyretin) levels in severe PE cases have been proposed as a potential circulating biomarker [38,39].

Genes linked to coagulation and lipid homeostasis may also be relevant. APOH, a key antigen in antiphospholipid syndrome (APS), highlights a possible intersection between autoimmune thrombophilia and early-onset PE via its effects on endothelial integrity [40]. Dysregulated lipid transporters such as APOA1, APOB, and AFP point to perturbed lipid handling and systemic inflammation in PE pathogenesis [41,42,43]. Genes linked to coagulation and lipid homeostasis may also be relevant [40]. APOH, a key antigen in antiphospholipid syndrome (APS), highlights a possible intersection between autoimmune thrombophilia and early-onset PE via its effects on endothelial integrity [40]. Meanwhile, PLG and its role in regulating epithelial sodium channels (ENaCs) may contribute to fluid retention and hypertensive features in PE [40,44]. 

AXLi also reversed several genes affecting trophoblast and immune function. LGALS14 and LGALS13 (PP13), which promote trophoblast invasion, are typically reduced early in pregnancies that later develop PE [45,46]. The re-expression of GATA1, an immune transcription factor, may reflect a broader immunomodulatory rebalancing, supported by animal data that shows symptom relief upon its suppression [47].

Other genes may relate to mitochondrial function, volume regulation, and innate immunity. LRRC8A, involved in cell volume control [48], shows increased placental activity during normal pregnancy, while FAM111A, which responds to DNA replication stress, has been implicated in placental pathology [49]. Recovery of TXN2, a mitochondrial thioredoxin, suggests a possible restoration of redox balance [50], and rescue of AKNA and DDX60 may reflect improved transcriptional and antiviral readiness in the placenta [51,52,53].

### 4.5. Implications of Gas6-AXLi Signaling

**Figure 8:**
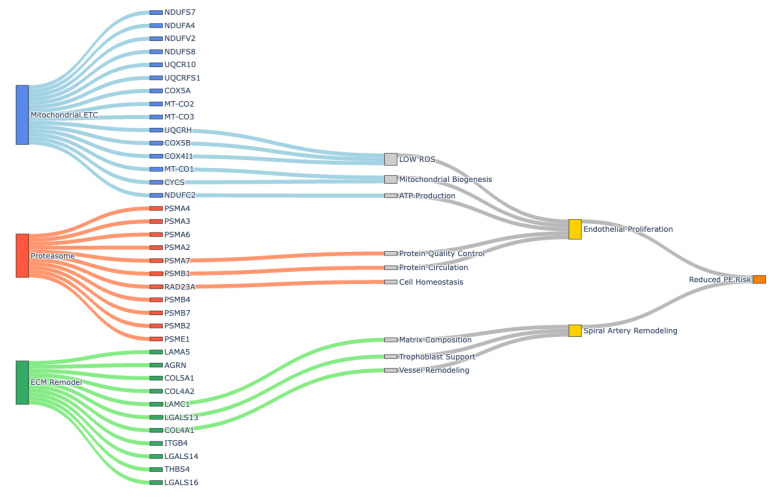
Integrated Multi-Layered Sankey Diagram of Gas6/AXL-Targeted Pathway Restoration: Conceptual Sankey diagram illustrating a potential mechanism to restore angiogenic pathways following treatment with AXLi. The diagram captures three primary biological systems (ETC, Proteasome–Ubiquitin System, and ECM Remodeling) at the leftmost layer, each connecting to their respective gene targets.

AXL inhibition could promote a reversed PE phenotype by reducing signs and symptoms (Figure 8). Specifically, AXL inhibition reduces angiogenesis through multiple coordinated mechanisms: restoring mitochondrial electron transport chain function, enhancing ATP availability for endothelial cell proliferation and migration, and reducing ROS to prevent endothelial damage. This treatment upregulated 21 proteasome subunit genes, enhancing protein quality control critical for VEGF signaling and endothelial survival. The treatment also normalized ECM components such as collagen and laminin, restoring MMP–TIMP equilibrium for controlled vessel sprouting. In PE, AXLi at least partially ameliorates impaired spiral artery remodeling by enhancing trophoblast invasion and vascular transformation, which is supported by increased mitochondrial biogenesis and reduced oxidative stress.

### 4.6. Limitations and Future Directions

These results suggest that suppressing Gas6-AXL signaling, whether through the withdrawal of Gas6, direct AXL inhibition, or both, can reverse some PE-like features through mitochondrial restoration, normalization of the extracellular matrix, proteolytic clearance, and ribosomal repair. This finding aligns with prior studies showing elevated soluble AXL (sAXL) levels in the plasma of patients with severe PE [19]. Key limitations include using a rat model, which focuses on late-gestation outcomes, and the potential for off-target effects of the AXL inhibitor R428. We believe that additional wet laboratory experiments are justified to elucidate further the underlying molecular mechanism of PE and the continued development of effective therapeutics.

## Figures and Tables

**Table 1:** Log2FC and FDR for Gas6 vs. Control (denoted as Gas6) comparison and Gas6-AXLi vs. Gas6 (denoted as AXLi) comparison.

Gene	Log2FC Gas6	FDR Gas6	Log2F AXLi	FDR AXLi
ALDH3B2	−8.854	8.991 × 10^−4^	8.285	0.007
TTR	−2.573	0.006	3.567	1.751 × 10^−4^
APOA1	−2.471	7.537 × 10^−4^	3.119	5.955 × 10^−5^
APOH	−2.940	0.003	3.312	0.004
APOA2	−2.392	0.019	3.246	0.003
APOB	−2.355	0.038	2.441	0.011
AFP	−2.351	0.006	2.947	9.469 × 10^−4^
PLG	−1.422	0.024	2.078	0.010
MTTP	−1.828	0.007	1.718	0.043
LGALS14	−1.427	2.510 × 10^−6^	1.700	3.517 × 10^−7^
LGALS13	−1.427	2.510 × 10^−6^	1.700	3.517 × 10^−7^
LGALS16	−1.427	2.510 × 10^−6^	1.700	3.517 × 10^−7^
PDZK1	−1.327	0.006	1.260	0.003
GATA1	−1.115	0.022	1.174	0.004
LRRC8A	1.000	0.006	−1.040	4.410 × 10^−5^
FAM111A	3.780	1.910 × 10^−23^	1.730	0.004
P2RX5	1.850	0.032	1.930	0.004
APBA1	2.100	0.014	−2.000	0.004
NIBAN2	2.110	0.040	2.510	2.420 × 10^−5^
FBXO33	5.880	0.220	−4.510	0.006
TXN2	7.860	0.026	−0.400	0.001
AKNA	8.690	7.790 × 10^−8^	−8.710	6.930 × 10^−9^
DDX60	8.900	1.500 × 10^−4^	−8.930	1.550 × 10^−5^

## Data Availability

All processed data discussed are presented within the article. The raw reads can be found in https://zenodo.org/records/16415649 (accessed on 7 August 2025).
